# Intense or malicious? The decoding of eyebrow-lowering frowning in laughter animations depends on the presentation mode

**DOI:** 10.3389/fpsyg.2014.01306

**Published:** 2014-11-18

**Authors:** Jennifer Hofmann

**Affiliations:** Personality and Assessment, Institute of Psychology, University of ZurichZurich, Switzerland

**Keywords:** FACS, Darwin, Duchenne Display, laughter, intensity, avatar

## Abstract

Joyful laughter is the only laughter type that has received sufficient validation in terms of morphology (i.e., face, voice). Still, it is unclear whether joyful laughter involves one prototypical facial-morphological configuration (Duchenne Display and mouth opening) to be decoded as such, or whether qualitatively distinct facial markers occur at different stages of laughter intensity. It was proposed that intense laughter goes along with eyebrow-lowering frowning, but in decoding studies of pictures, these “frowns” were associated with perceived maliciousness rather than higher intensity. Thus, two studies were conducted to investigate the influence of the presentation mode (static, dynamic) and eyebrow-lowering frowning on the perception of laughter animations of different intensity. In Study 1, participants (*N* = 110) were randomly assigned to two presentation modes (static pictures vs. dynamic videos) to watch animations of Duchenne laughter and laughter with added eyebrow-lowering frowning. Ratings on the intensity, valence, and contagiousness of the laughter were completed. In Study 2, participants (*N* = 55) saw both animation types in both presentation modes sequentially. Results confirmed that the static presentation lead to eyebrow-lowering frowning in intense laughter being perceived as more malicious, less intense, less benevolent, and less contagious compared to the dynamic presentation. This was replicated for maliciousness in Study 2, although participants could potentially infer the “frown” as a natural element of the laugh, as they had seen the video and the picture. Thus, a dynamic presentation is necessary for detecting graduating intensity markers in the joyfully laughing face. While this study focused on the decoding, future studies should investigate the encoding of frowning in laughter. This is important, as tools assessing facially expressed joy might need to account for laughter intensity markers that differ from the Duchenne Display.

## INTRODUCTION

This study investigated the perception of the progression of facial morphological features during joyful laughter at different stages of laughter intensity. Whereas most past studies focused on distinguishing qualitatively different types of laughter, this study concentrated on one of the few commonly agreed on types of laughter: joyful or amusement laughter (e.g., Darwin, 1872; Szameitat et al., 2009; Wildgruber et al., 2013). The main aim of the study was to examine whether joyful laughter is decoded best as a single morphological configuration with a given set of facial features of similar intensity (e.g., Duchenne Display, the joint and symmetric contraction of zygomatic major muscle and orbicular is oculi pars orbitalis muscle; Ekman et al., 1990) plus a laughter-related vocalization and mouth opening (see Ruch and Ekman, 2001), or whether qualitatively distinct facial markers (like eyebrow-lowering frowning) can occur at different stages of laughter intensity that do not diminish the perception as joyful.

The notion of a progression of facial features at increasing stages of (joyful) laughter intensity was already described by Darwin (1872). From a current perspective, Darwin (1872) described joyful laughter to entail a Duchenne Display with an open mouth, possibly jaw relaxation, and raised lips (converging with more recent definitions of joyful laughter, see Keltner and Bonanno, 1997; Ruch and Ekman, 2001), as well as eyebrow-lowering frowning in the case of *intense* joyful laughter. Not only Darwin, but also his contemporary writers delivered extensive descriptions of laughter types and their facial expressions (see Ruch et al., 2013).

A recent study of these historic illustrations of laughter (stemming from Darwin’s coevals, Ruch et al., 2013) investigated the en– and decoding of joyful and intense joyful laughter in static visual illustrations. The examination of facial features in 18 illustrations with the Facial Action Coding System (FACS, Ekman et al., 2002) and the decoding by laypeople showed that illustrations involving a Duchenne Display were perceived as joyful – irrespective of their initial laughter classification by the original authors, and the perception of joyfulness was linked to the intensity of the orbitalis oculi pars orbitalis activity (Action Unit [AU6]; Cheek Raiser in the FACS). In intense laughter, the intensity of the zygomatic major muscle contraction (AU12, Lip Puller) predicted the perception of intensity, but not frowning that lowers the eyebrows (AU4; Brow Lowerer; consisting of the action of the m. depressor glabellae, m. depressor supercilli, and m. corrugator). In fact, eyebrow-lowering “frowning” present in intense laughter illustrations seemed to be antagonistic to the perception of joy. Ruch et al. (2013) concluded that only the Duchenne Display could be reliably recognized, and that the perception of intensity was linked to the intensity of the Duchenne markers, not frowning in the eyebrow region.

In contrast, a recent approach utilizing videos (motion capture data of facial activity) of joyful laughs found supporting evidence for Darwin’s claim: The automatically detected eyebrow-lowering frowning movements in joyful laughter expressions were correlated to ratings of perceived laughter intensity (Niewiadomski et al., 2012). Therefore, the current approach aims at testing the two postulates on the facial features and perception of intense laughter. It was tested whether a) eyebrow-lowering frowning may be involved in intense laughter and predicts the perception of laughter intensity (see Darwin, 1872; Niewiadomski et al., 2012, or b) the perception of laughter intensity is linked to the intensity of Duchenne markers of the joyful laughter expression (e.g., Ruch et al., 2013), whereas eyebrow-lowering frowning leads to the perception of maliciousness (due to its frequent occurrence in negative emotions, such as anger, see Ruch et al., 2013). This is of relevance, because if joyful laughter is not a unitary facial configuration (i.e., Duchenne laughter) and different stages of intensities entail distinct morphological features, studies that do not take the intensity of laughter into account may come to mixed conclusions (especially if certain intensity-related facial features alter the qualitative decoding for the different stages of intensity). The notion that single qualitative features can alter the decoding of a laugh has already been tested and confirmed for laughter acoustics (see Bachorowski and Owren, 2001; Kipper and Todt, 2003), but not for the facial expression of laughter. Similarly to acoustical features, single facial features occurring at certain levels of laughter intensity might alter the perception and behavioral responses of individuals.

Thus, laughter stimuli are needed to test whether the addition of eyebrow-lowering frowning leads to an altering of the perception of the laughter (while keeping all other features constant). Concerning laughter stimuli utilized in recent laughter investigations, en- and decoding studies of laughter features were typically conducted on selected laughter types and separately for modalities of the face (encoding: see Keltner and Bonanno, 1997; decoding: e.g., Ruch et al., 2013) and the voice (encoding: Bachorowski and Owren, 2001; Bachorowski et al., 2001; decoding: Kipper and Todt, 2003; Szameitat et al., 2009; Sauter et al., 2010; Scott et al., 2010; Wildgruber et al., 2013). One exception is the study by Sestito et al. (2013) who investigated the decoding of audio-visual laughter stimuli. They found that the correct decoding of laughter was high above chance rate and that in audio-visual incongruent stimuli, the visual modality was prioritized in the decoding over the acoustic dimension. They also reported that rapid and congruent mimicry toward laughter stimuli (EMG measured zygomatic major muscle activity) occurred. Studies on the en-and decoding of laughter typically included the encoding of posed laughs and the decoding of natural, as well as posed laughter^1^. A limitation of many of these studies is that the tested stimuli are under little experimental control: posed laughs are stereotypical and might represent display rules and cultural conventions. In contrast, spontaneous laughs are highly variable and may be influenced by a personal laughter signature (Ruch and Ekman, 2001 and also Philippon et al., 2013). Furthermore, naturally occurring laughs might entail markers of up- or down regulation or the distortion through social display rules. Thus, stimuli are needed that allow for controlling the occurring facial features in a systematic manner.

In terms of stimulus presentation, dynamically presented stimuli are likely to outperform static stimuli (i.e., pictures, illustrations) in recognition rates and contagiousness. Indeed, recent studies provided support for the notion that synthetic and human dynamic faces have stronger emotional effects and entail information that static pictures do not contain (e.g., Wehrle et al., 2000; Rubenstein, 2005; Sato and Yoshikawa, 2007; Horstmann and Ansorge, 2009; Krumhuber et al., 2009). Thus, static stimuli do not reveal the important dynamics for a decoding task, and natural laughs as well as posed laughs are difficult to control (i.e., no control over “laughter signature,” display rules, regulation). As the two postulates on the interpretation of eyebrow-lowering frowning in intense laughter stemmed from both presentation modes (static and dynamic), they should be tested simultaneously.

As an alternative to the stimuli used in past studies, the lack of control could be overcome by utilizing the captured data of naturally occurring laughs and animate them onto a virtual character. Video animations overcome limitations that auditory clips and pictures have: auditory stimuli lack the visual context and still pictures lack the information on onset, apex, and offset of a laugh, including timing. Additionally, utilizing a virtual character allows for a control of the facial features in a standardized way (e.g., Krumhuber et al., 2009). Next to an original animation of laughter expressions, it is also possible to modify single Action Units (AUs; Ekman et al., 2002) for the testing of hypotheses (in this case, the adding of eyebrow-lowering frowning to intense laughter). Whereas also single auditory features could be manipulated (see e.g., Kipper and Todt, 2001, 2003), the current approach focused on the manipulation of facial features. A freely available virtual character was utilized, driven by high-level facial behavior descriptions based on the FACS (Niewiadomski et al., 2009, 2011). This allowed for a controlled variation of facial configurations (see Boker et al., 2009) and their intensity (Niewiadomski et al., 2012).

## AIMS OF THE STUDY

The current study investigated the perception of the progression of facial morphological features during joyful laughter at different stages of laughter intensity. In particular, the role of eyebrow-lowering frowning on the perception of differently intense joyful laughter was investigated, testing two competing postulates. In two decoding studies, laughter samples were presented as static (picture) and dynamic (video) animations of a virtual agent. Whereas the dynamic stimuli provided the natural dynamics of the laugh in its facial features, the static stimuli did not, expectedly leading to an altering of the decoding information (i.e., frowning being perceived as malicious). In Study 1, participants (assigned to either static or dynamic presentation) rated laughter animations of Duchenne laughs and laughs with added frowning as intensity marker on several rating scales (i.e., intensity, maliciousness, benevolence, contagiousness).

Firstly, it was expected that the dynamic presentation would lead to higher perceived intensity in both, intense Duchenne laughs and laughs with eyebrow-lowering furrows, compared to the static presentation (H1a), as it was shown that synthetic dynamic stimuli generally outperform static stimuli in ratings of intensity (e.g., Kätsyri and Sams, 2008; for a review see Krumhuber et al., 2013). Basing on the findings by Ruch et al. (2013), an interaction between the presentation mode and the type of laughter animation was expected: It was assumed that the difference in rated intensity between the dynamic vs. static presentation mode would be larger for the intense laughs with eyebrow-lowering furrows than the intense Duchenne laughs (H1b). For the perception of maliciousness, the reverse influence of presentation mode on the perception of maliciousness was expected: It was assumed that the static presentation mode would lead to higher perceived maliciousness in intense laughs with eyebrow-lowering furrows as compared to intense Duchenne laughs (in accordance with the findings of Ruch et al., 2013), while the dynamic presentation would lead to lower rated maliciousness for both animation types (H2). Regarding the perceived benevolence (H3) and contagiousness (H4), it was again assumed that an interaction between the presentation mode and the type of laughter animation would occur for the respective ratings of intense laughter, convergent to the expectations on rated intensity.

In Study 2, the laughter animations were presented in both presentation modes (static and dynamic) in a repeated measures design to an independently collected sample of participants. It was aimed to extend the findings of Study 1 by testing the stability of the results while modifying two features of the design: firstly, individuals were presented with both stimuli types, first seeing the dynamic animations then the static pictures. Studies have shown that individuals can reproduce the temporal progression of emotion expressions from a set of static pictures (Edwards, 1998). Thus, it was assumed that participants transfer the knowledge on the intensity from the dynamic to the static stimuli similarly when seeing the dynamic stimuli first. This would consequently diminish the differences in perceived intensity between static and dynamic stimuli (H5). It was further assumed that participants “learn” to understand the eyebrow-lowering frown as a feature of intense laughter when seeing the dynamic stimuli and transfer this knowledge to the later presented static stimuli. In consequence, the rating bias of eyebrow-lowering frowning as being malicious should subsequently disappear in the rating of the presented static stimuli (H6). Secondly, while Study 1 utilized video-audio animations, Study 2 utilized toneless video-only animations, to control for effects of the laughter sounds on the ratings.

Moreover, the varying facial features might impact on decoders differently, in the perception of intensity, valence, and contagiousness. While laughter had been claimed to be universally recognized as a signal of joy and is one of the non-verbal stimuli with the most positive valence (Sauter et al., 2010; Lima et al., 2014), individual differences in the perception of laughter were found. More specifically, the fear of being laughed at (gelotophobia) predicts the systematic misperception of good-natured laughter and teasing (see Ruch et al., 2014 for a review). Thus, high scores on the measure for the fear of being laughed at were used as an exclusion criterion in the current studies.

## STUDY 1

### METHODS

#### Participants

The sample consisted of 143 English-speaking adults with a mean age of 31.01 years (SD = 10.80, range = 18 – 70; 67 males, 74 females, two did not indicate their gender). The gelotophobia scores ranged from 1.00 to 3.93 (*M* = 1.95, SD = 0.67). Out of the 143 participants, 110 indicated no fear of being laughed at (i.e., scores lower than 2.5 on the standard questionnaire measure for gelotophobia, the GELOPH<15>; Ruch and Proyer, 2008) and 33 participants exceeded the cut-off value for gelotophobia (>2.5). The 33 gelotophobes were excluded from subsequent analyses. From the remaining 110 participants, 60 were assigned to the static presentation and 50 to the dynamic presentation condition. A Chi-Square indicated no systematic drop-out from either condition, χ^2^(1) = 0.11, *p* = 0.535.

#### Materials

***Instruments.*** The GELOPH<15> (Ruch and Proyer, 2008) is the standard questionnaire for the assessment of gelotophobia (e.g., “when they laugh in my presence I get suspicious”) consisting of 15 items in a four-point answer format (1 = strongly disagree to 4 = strongly agree). In the current sample, the Cronbach’s alpha was high (α = 0.93). Mean scores ≥ 2.50 in the GELOPH<15> indicate at least a slight expression of gelotophobia (Ruch and Proyer, 2008).

In the Laughter Evaluation Questionnaire (LEQ), participants are asked to rate items to each presented laughter stimulus. Participants rate the perceived dynamics (“how dynamic are the facial features of the laugh?”), intensity (“how intense is the laugh?”), valence (“how malicious is the laugh?”, “how benevolent is the laugh”), and contagiousness of the laugh (“how contagious is the laugh?”). All items are answered on a five point Likert scale ranging from 1 = not at all to 5 = extremely.

***Laughter stimuli.*** For the stimuli selection and synthesis of laughs to animations, several steps were necessary. Firstly, suitable joyful laughs were identified (six clips from the AVLC corpus; Urbain et al., 2009). Three intensity levels were considered (low, medium, high; see Niewiadomski et al., 2012), as changes in the perception were expected for the different stages of laughter intensity. The laughs where synthesized onto a virtual agent twice: Once in the original configuration (Duchenne laughs) and once modified by adding an eyebrow-lowering frowning action (equivalent to the AU4 in the FACS) parallel to the action of the AU12. Moreover, 12 dynamic stimuli (videos) and 12 static stimuli (pictures) were derived from the virtual agent animations, leading to 24 laughter stimuli. The stimuli generation is described in the following sections.

***Original clips.*** The freely available Audio Visual Laughter Cycle (AVLC) corpus (Urbain et al., 2009) contains about 1,000 spontaneous audio-visual joyful laughter episodes (amusement elicited through watching funny videos; 24 participants; 4–82 laughter episodes per participant). The subjects were filmed in a head close up, frontal view, with the shoulders slightly visible. All clips were annotated for the perceived laughter intensity using a five-point Likert scale from 1 = low intensity to 5 = high intensity (for details see Niewiadomski et al., 2012). For the current study, six videos of joyful laughter bouts of one female participant were chosen (two low intensity, two medium intensity, and two high intensity laughs; coded with the FACS for the occurrence of AU 4, 6, 7, 12, as well as 25/26/27 by the author and control-coded by a further certified coder). All laughs were voiced laughs (as differences in valence ratings have been reported for voiced vs. unvoiced laughs, e.g., Bachorowski and Owren, 2003), and head movements to the side and back. The durations of the clips ranged between 2–7 s.

***Dynamic stimuli: video animations.*** From the original joyful laughs, 12 animations were generated. The animations were done by the Facial Animation Parameter (FAP) animation method and coded for their AU content by two trained FACS coders. The animations were displayed by a freely available avatar (for technical details see Qu et al., 2012). The avatar was presented in a head close up, frontal view. No torso movements were animated. Two types of animations were generated: (a) the original Duchenne laugh, and (b) laughs with eyebrow-lowering furrows (with added AU4 as intensity marker). The AU4 action was matched to the timely development and intensity of the AU12.

**FIGURE 1:**
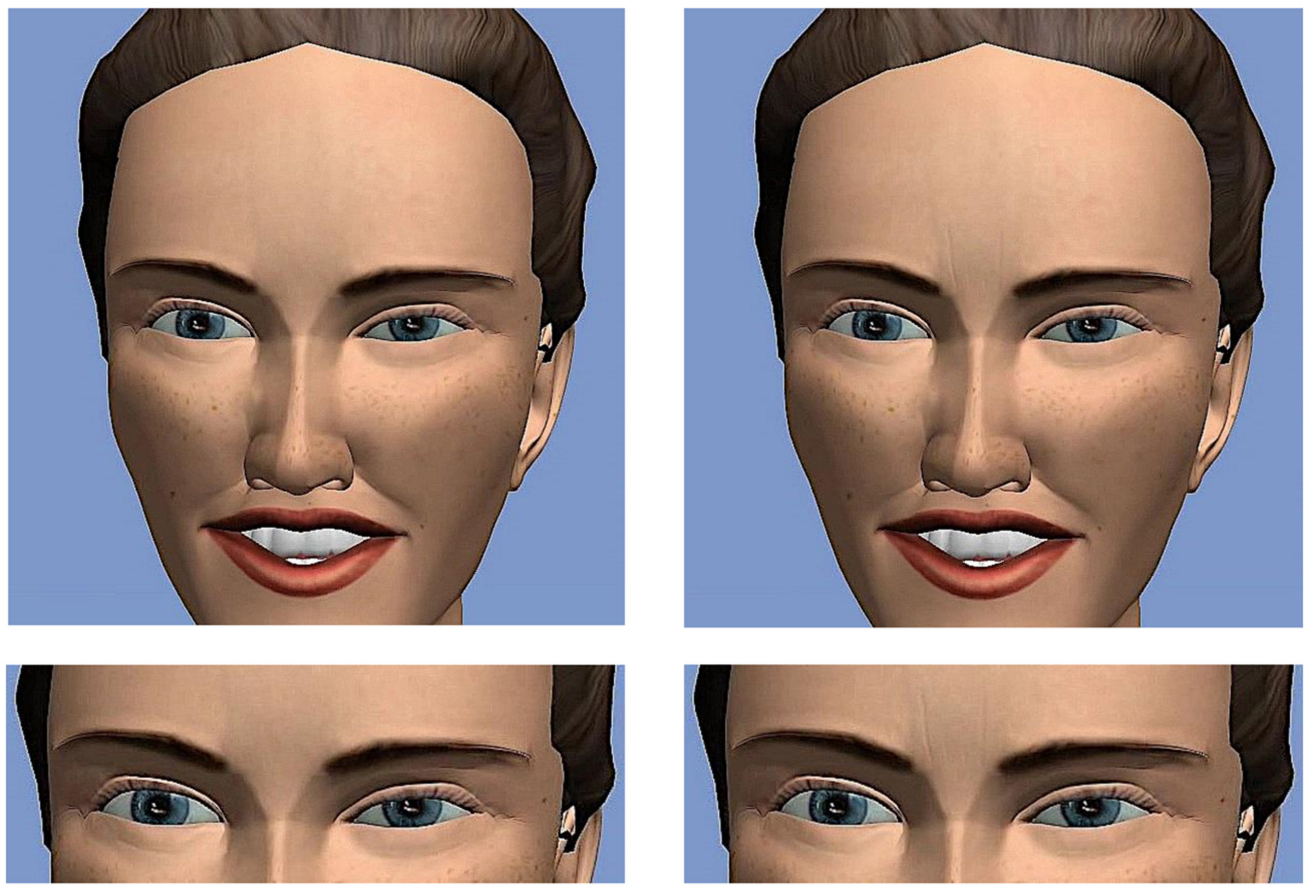
**Top row: example of one static laugh stimulus, representing the apex of a Duchenne laugh **(left)** and the same laugh with added vertical wrinkles indicating the eyebrow-lowering frown **(right)**. **Bottom row:**** close up on the vertical wrinkles indicating eyebrow-lowering frowning on the virtual agent.

***Static stimuli: pictures of the apex of the laughter event.*** To generate the static stimuli from the 12 laughter animations, the apex of the laughter event (operationalized by the apex of the AU12) was determined by a trained FACS coder and still pictures were derived at this time point. **Figure 1** shows one example of a static laughter stimulus in both animation types (Duchenne laugh and laugh with eyebrow-lowering furrows).

The top row of **Figure 1** shows an example of one static laugh stimulus, representing the apex of a Duchenne laugh (left) and the same laugh with wrinkles indicating the eyebrow-lowering frown (right side). On the bottom row, a close up on the eyebrow region is given, where the added wrinkles can be seen on the right. The static stimuli were presented on single web pages (with the ratings beyond the picture).

#### Design

Participants were randomly assigned to one of two presentation conditions: either judging laughter stimuli of different intensities in static (picture) or dynamic (full video animation) stimuli. Thus, participants judged 12 laughter stimuli in two variations: six Duchenne laughs, and six laughs with eyebrow-lowering furrows. The 12 stimuli were presented in random order. Before the experimental task, participants completed the GELOPH<15>.

#### Procedure

Participants were given a link to an online survey where they received the general instructions to the study. First, they filled in demographic variables (age, gender, education level) and the GELOPH<15>. Second, the instructions to the study were presented. Participants were instructed to wear headphones during the study and to complete the study in a quiet environment. Also, participants were informed about the technical requirements of the web browser (i.e., allowing for cookies, sound) which would be necessary to complete the study smoothly. Participants were informed that they would be presented with animations/pictures of a laughing avatar and that their task was to rate the laughs on several scales (intensity, maliciousness, benevolence, contagiousness). They were made aware that some of the laughter animations/pictures might look similar, as the animations/pictures were chosen from a big pool of laughter examples and were randomly assigned to them. Furthermore, participants were told that the laughs contain audio, but that they should focus on the facial features. Third, the rating of the laughter stimuli started. Participants could see the animations/pictures only once and were then asked to rate the facial expression of the laughter. They had to rate all the items before seeing the next animation/picture. All animations and pictures were presented in random order. After completing the study, participants were thanked for their participation and debriefed. All participants stayed anonymous at all times. The study was approved by the local Ethics Committee (Institute of Psychology, University of Zurich) and followed the APA standards.

### RESULTS

#### Manipulation of the presentation mode

To investigate whether the experimental manipulation (presentation mode) was successful, the assigned dynamics to the laughter stimuli were analyzed. A repeated measures analysis of variance (ANOVA) with the presentation mode (dynamic vs. static) as between subjects factor, the type of animations (Duchenne laughs, laughs with eyebrow-lowering furrows) as repeated measures, and the degree of perceived dynamics as dependent variable was computed. As expected, the dynamic presentation mode (Duchenne: *M* = 2.74, SD = 0.73; laughs with eyebrow-lowering furrows: *M* = 2.64, SD = 0.84) led to higher ratings of dynamics compared to the static presentation mode (Duchenne: *M* = 2.29, SD = 0.77; laughs with eyebrow-lowering furrows: *M* = 2.26, SD = 0.82), *F*(1,94) = 7.70, *p* = 0.007, η_p_^2^ = 0.076. In line with the expectations, there was no interaction with the two animation types, *F*(1,94) = 0.03, *p* = 0.866, nor an effect for the type of animation, *F*(1,94) = 2.39, *p* = 0.125.

#### The perception of intensity, maliciousness, benevolence, and contagiousness

**FIGURE 2:**
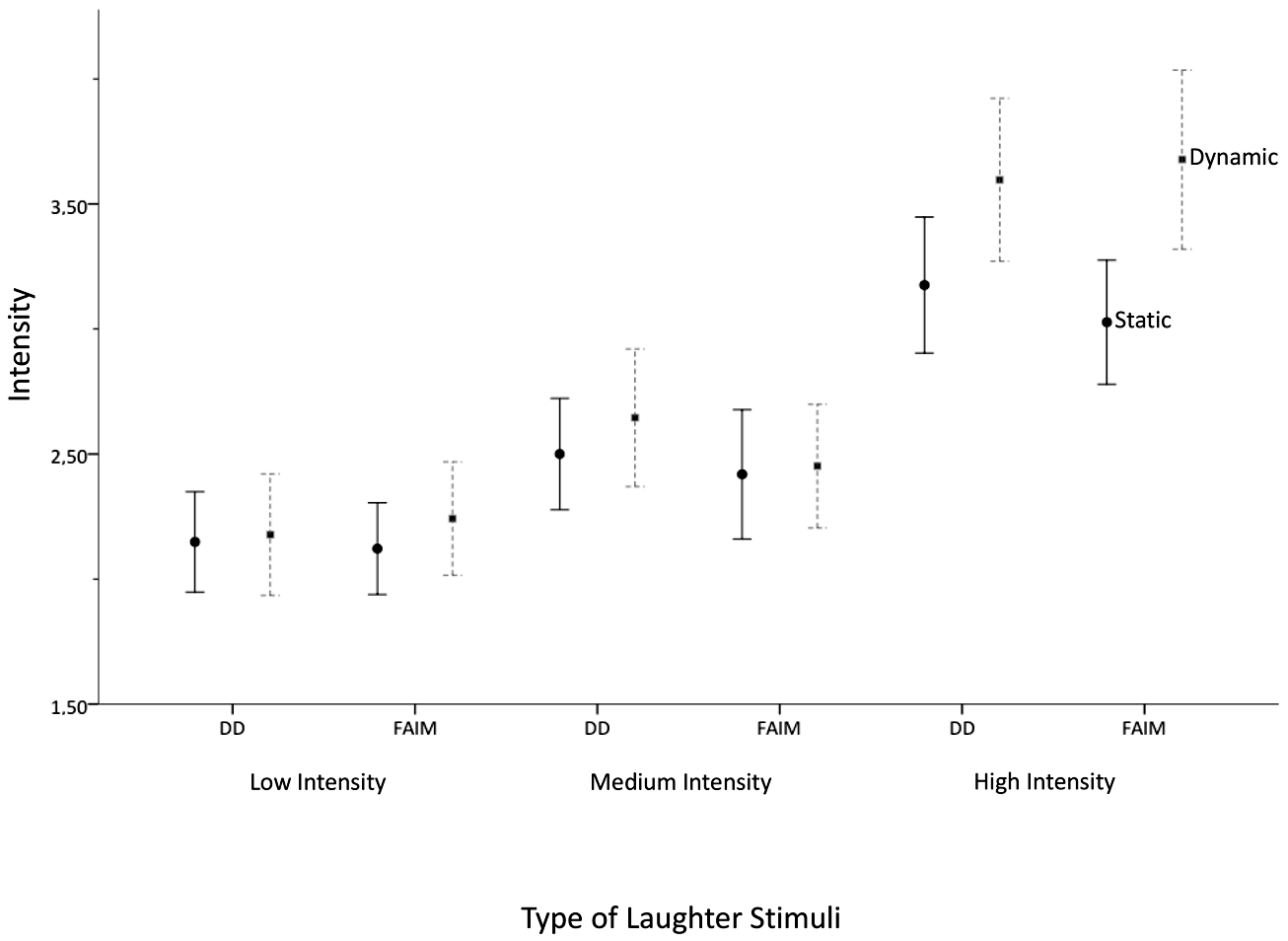
**Mean intensity ratings and standard error bars (2±) for Duchenne laughs and laughs with added eyebrow-lowering furrows (FAIM) in three intensities (low, medium, high)**.

Next, the four hypotheses on the influence of presentation mode, stimulus type, and laughter-display intensity on the ratings of perceived laughter intensity, maliciousness, benevolence, and contagiousness are reported. First, the ANOVA analysis on the perceived intensity of laughter is reported (H1a and H1b). Hypothesis H1a stated that the dynamic presentation would lead to higher perceived intensity in both, intense Duchenne laughs and laughs with eyebrow-lowering furrows, compared to the static presentation. Hypothesis H1b assumed that the difference in rated intensity between the dynamic vs. static presentation mode would be larger for the intense laughs with eyebrow-lowering furrows than the intense Duchenne laughs. **Figure 2** shows the means and SD for the perceived intensity in the different laughter stimuli (low, medium and high intensity Duchenne laughs and laughs with eyebrow-lowering furrows).

**FIGURE 3:**
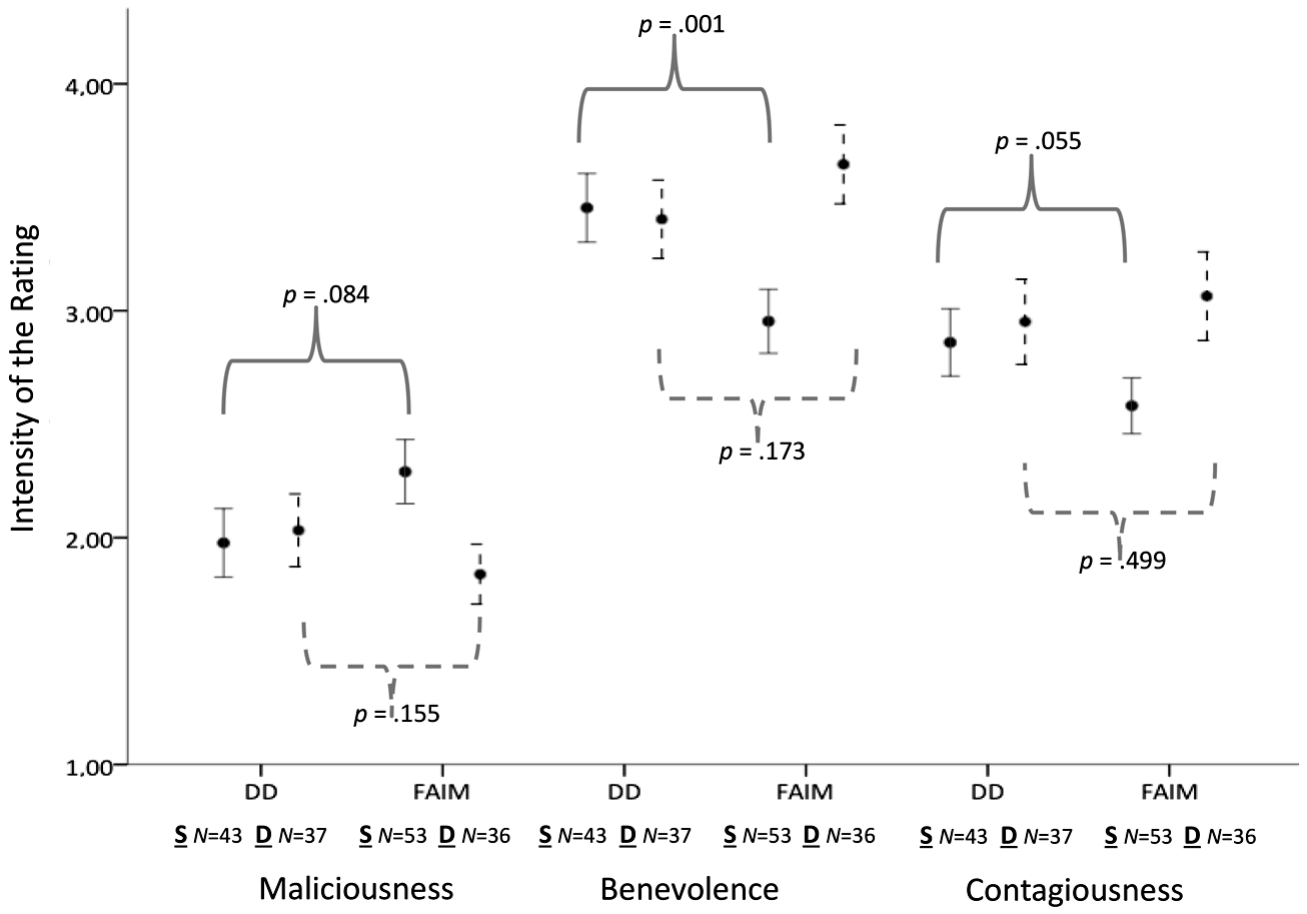
**Within-subject changes for the ratings of intense laughs (Duchenne laughs and laughs with eyebrow-lowering furrows).** Full lines = Static presentation condition. Dotted lines = Dynamic presentation condition. DD = Intense Duchenne laughs. FAIM = Intense laughs with added eyebrow-lowering frowning as intensity marker. S, Static; D, Dynamic.

A repeated measures ANOVA with the degree of perceived intensity as dependent variable, the presentation mode (static vs. dynamic) as factor, and the type of animations (Duchenne laughs and laughs with eyebrow-lowering furrows in low, medium, and high intensity) as repeated measures was computed. **Figure 2** shows that the rated intensity differed for the laughter stimuli, *F*(5,330) = 57.92, *p* = 0.001, η_p_^2^ = 0.467. All *post* hoc pairwise comparisons (Bonferroni corrected) indicated that the high intensity laughs yielded the highest intensity ratings (Duchenne laughs and laughs with eyebrow-lowering furrows). They were followed by the medium laughs being lower than the high intense laughs, but higher than the low intensity laughs, validating the a priori assigned intensity levels. The main effect “presentation mode” confirmed H1a by showing that the dynamic presentation lead to generally higher intensity ratings than the static presentation, *F*(1,66) = 3.55, *p* = 0.036, η_p_^2^ = 0.064. In line with H1b, the effect was qualified by an interaction between the type of animation and the presentation mode (static vs. dynamic), *F*(5,330) = 2.94, *p* = 0.013, η_p_^2^ = 0.043. *Post hoc* comparisons indicated that the presentation modes led to differences in the rating of the high intensity laughs with eyebrow-lowering furrows, *p* = 0.001 level (see **Figure 2**).

Next, H2 to H4 were tested. Means and SD for the maliciousness ratings (H2), benevolence (H3), and contagiousness (H4) ratings are reported in **Table 1**.

**Table 1:** Means and standard deviations of the valence and contagiousness ratings.

		Presentation mode					
Rating		Static		Dynamic			
		*M*	SD	*M*	SD	*F* (df1, df2)	*p*
Maliciousness	DD Low	1.79	0.83	2.08	0.75		
	DD Medium	2.15	1.02	2.21	0.86		
	DD High	1.97	0.99	2.05	0.92	0.19 (1,80)	0.721
	FAIM Low	2.00	0.79	2.46	1.04		
	FAIM Medium	2.24	0.93	2.29	0.82		
	FAIM High	2.41	1.05	1.83	0.69	5.51 (1,89)	0.020
Benevolence	DD Low	2.70	0.68	2.80	0.82		
	DD Medium	2.85	0.78	3.03	0.80		
	DD High	3.45	0.99	3.31	1.05	0.63 (1,80)	0.802
	FAIM Low	2.28	0.79	2.65	0.82		
	FAIM Medium	2.40	0.85	2.74	0.79		
	FAIM High	2.79	0.98	3.69	0.93	7.03 (1,80)	0.009
Contagiousness	DD Low	1.98	0.68	1.84	0.71		
	DD Medium	2.12	0.77	2.38	0.91		
	DD High	2.86	0.97	2.82	1.16	0.02 (1,80)	0.880
	FAIM Low	2.00	0.72	1.79	0.84		
	FAIM Medium	2.13	0.88	1.97	0.84		
	FAIM High	2.43	0.89	3.05	1.03	9.07 (1,89)	0.003

*Static = Picture of the apex of the laughter event. Dynamic = Full video animation. DD = Duchenne laugh. FAIM = Laugh with added frown as intensity marker. Low = Low Intensity. Medium = Medium Intensity. High = High Intensity.*

**Table 1** shows the ratings of maliciousness in the different presentation modes. Hypothesis H2 assumed that the static presentation mode would lead to higher perceived maliciousness in intense laughs with eyebrow-lowering furrows as compared to intense Duchenne laughs, while the dynamic presentation would lead to lower rated maliciousness for both animation types. To investigate H2, a repeated measures ANOVA with the presentation mode (static vs. dynamic) as factor, the type of animation as repeated measure (low, medium, and high intensity Duchenne laughs and laughs with eyebrow-lowering furrows) and the degree of maliciousness as dependent variable was computed. H2 was confirmed: the interaction effect of the presentation mode by laughter stimulus indicated a disordinal interaction *F*(3.986, 263.078) = 3.54, *p* = 0.008, η_p_^2^ = 0.051 (Greenhouse-Geisser corrected degrees of freedom, ε = 0.797, for violations of sphericity assumptions). In line with H2, the *post hoc* tests showed that for the case of intense laughs with eyebrow-lowering furrows, the static presentation mode led to higher perceived maliciousness compared to the dynamic presentation mode, *F*(1,89) = 5.51, *p* = 0.020, η_p_^2^ = 0.083^2^.

For the ratings of benevolence (H3) and contagiousness (H4), the means and standard deviations are reported in **Table 1**. Two repeated measures ANOVAs with the presentation mode (static vs. dynamic) as factor, the type of animation as repeated measure (two high intensity laughs of the Duchenne and laughs with eyebrow-lowering furrows) and the degree of benevolence and contagiousness respectively as dependent variable were computed. Hypothesis H3 assumed that an interaction between the presentation mode and the type of laughter animation would occur for the rating of benevolence in intense laughter, convergent to the expectations on rated intensity. For the benevolence ratings (H3), the expected interaction between the presentation mode and the type of animation occurred, *F*(1,77) = 7.11, *p* = 0.009, η_p_^2^ = 0.085, showing that the dynamic presentation led to higher benevolence ratings of the laughs with eyebrow-lowering furrows compared to the static presentation mode; *F*(1,95) = 7.03, *p* = 0.009, η_p_^2^ = 0.069. Expectedly, the presentation mode did not have an impact on perceived benevolence the Duchenne laugh, *F*(1,80) = 0.63, *p* = 0.802.

Hypothesis H4 assumed that an interaction between the presentation mode and the type of laughter animation would occur for the rating of contagiousness in intense laughter, convergent to the expectations on rated intensity. For the ratings of contagiousness (H4), the expected interaction between the presentation mode and the type of animation occurred, confirming H4, *F*(1,72) = 3.08, *p* = 0.044, η_p_^2^ = 0.041. Whereas both presentation modes led to equally high contagiousness of the intense Duchenne laughs, the dynamic presentation mode led to higher contagiousness in the laughs with eyebrow-lowering furrows than the static presentation; *F*(1,89) = 9.07, *p* = 0.003, η_p_^2^ = 0.094.

### DISCUSSION

Study 1 showed that the presence of eyebrow-lowering frowning only led to misperceptions of intense laughter as being less intense (H1b), more malicious (H2), less benevolent (H3), and less contagious (H4), when the laughter was presented *statically* compared to dynamic presentations. Although the effects on the ratings were small, they helped to clarify the emergence of the two postulates on the perception of eyebrow-lowering frowning as a marker of laughter intensity. Both former findings (Niewiadomski et al., 2012; Ruch et al., 2013) are correct for their respective method of presentation (static vs. dynamic). Thus, eyebrow-lowering frowning (which may occur in intense laughter) is not detrimental to the perception of intensity when the stimuli contain the full dynamic information, but lead to higher perceived maliciousness when the movement information is missing.

In general, there was a linear increase in perceived intensity with the a priori stages of intensity in the animations, but the interaction effect showed that the high intensity laughs (both, the Duchenne laughs and laughs with eyebrow-lowering furrows) were perceived more intense when presented dynamically, confirming H1a. The dynamic information increased the decoding performance of the raters, as the intensity ratings showed a trend of decreasing in laughs with a frown in the static presentation mode. Also, the presence of eyebrow-lowering frowning in intense laughter did not hinder the perception of intensity in the dynamic presentation, indicating that frowning can occur in intense laughter (in line with H1b).

In line with H2, eyebrow-lowering frowning in intense laughter only led to increased perceived maliciousness when the stimuli were presented statically. Interestingly, additional frowning in low and medium intensity laughter led to higher ratings of maliciousness compared to Duchenne laughs in the dynamic presentation mode. This might be an indicator for the link between qualitative feature changes that are related to increasing laughter intensity: Darwin (1872) described frowning as a qualitative marker of high intensity laughter. Therefore, frowning in low/medium intensity laughter may be an intensity-incongruent feature. This might lead to an altering of the decoding (e.g., perception of an emotion blend), as reflected in the higher maliciousness ratings of the medium intense laugh (where the frowning naturally does not occur, due to the lower intensity).

With respect to the perception of benevolence and contagiousness, H3 and H4 were confirmed: The interaction between the animation type and the presentation mode in intense laughter was also found in the ratings of contagiousness and benevolence. The static presentation (compared to the dynamic presentation) led to lower benevolence and contagiousness for the intense laughs with eyebrow-lowering furrows, whereas no differences were found in the ratings of the intense Duchenne laugh. Thus, also for the ratings of valence and contagiousness, the presentation mode influenced the perception of the eyebrow-lowering frowning.

## STUDY 2

The aim of the second study was to follow-up two factors that might have influenced the findings of Study 1 in an independently collected sample: a) the effect of learning to recognize the eyebrow-lowering frown as a feature of intense laughter from the dynamic stimuli and b) the effect of the laughter sound in the dynamic stimuli. As a first modification of the design, participants were presented with both presentation modes (video animations first, then the pictures) to see whether the perception bias in static stimuli would persist if participants have had the dynamic information first. It was presumed that a “learning effect” would occur due to seeing the dynamic stimuli first and thus, would transfer this knowledge from the dynamic stimuli to the static (which in turn would diminish the perceived intensity and maliciousness differences). It was assumed that this would diminish the differences in perceived intensity between static and dynamic stimuli, as participants transfer the knowledge on the intensity form the dynamic to the static stimuli (H5). It was assumed that participants learn to understand the eyebrow-lowering frown as a feature of intense laughter when seeing the dynamic stimuli. As a consequence, the rating bias of frowning as being malicious should subsequently disappear in the rating of the presented static stimuli (H6). As a second modification, the video animations were presented without sound to diminish the effect the sound might have on the ratings. Thus, Study 2 focused on the perception of intensity and maliciousness in a repeated measures design of dynamic and static stimuli (visual only).

### METHOD

#### Participants

The sample consisted of 64 English-speaking adult participants with a mean age of 30.02 years (SD = 12.28, range = 18–73; 21 males, 43 females). The gelotophobia scores ranged from 1.00 to 3.27 (*M* = 1.83, SD = 0.55). Nine participants exceeded the cut-off value for gelotophobia and were excluded from the analyses.

#### Instruments and materials

As in Study 1, the gelotophobia score (GELOPH<15>) and the Laughter Evaluation Questionnaire (LEQ) were assessed. The Cronbach’s Alpha score for the GELOPH<15> in Study 2 was comparable to Study 1 (α = 0.93 for the GELOPH<15>). For the dynamic video animations, the same animations as in Study 1 were utilized but the *laughter sound* was omitted from the stimuli. The same static laughter picture material as in Study 1 was utilized.

#### Design and procedure

Participants were recruited over the university mailing list directed at students and online forums for an online survey. The general procedure was convergent to the procedure of Study 1: Participants were given a link to the online study, where they completed the demographic variables, the GELOPH<15> and received the instructions for the task. Then, all participants were presented with the Duchenne laughs and laughs with eyebrow-lowering furrows (low, medium, high intensities) in a repeated measures block design: First, the 12 dynamic soundless animations were presented in random order, followed by the 12 static animations also presented randomly. All ratings of the LEQ were obtained for the laughter stimuli, but the current analyses focused on the evaluation of the ratings of intensity and maliciousness. After answering all questions, participants were thanked for their participation and debriefed.

### RESULTS

First, H5 on the influence of the repeated presentation of dynamic and static stimuli on the perception of rated laughter intensity was investigated. Hypothesis H5 stated that participants transfer the knowledge on the intensity from the dynamic to the static stimuli similarly when seeing the dynamic stimuli first. This would consequently diminish the differences in perceived intensity between static and dynamic stimuli. For the intensity ratings, means and standard deviations are reported in **Table 2**.

**Table 2:** Means and SDs for the intensity and maliciousness ratings, and ANOVA results.

			Intensity					Maliciousness		
Presentation	Animation		Low	Medium	High	*F* (df1,df2)	Low	Medium	High
Dynamic	DD	M	1.98a	2.44b	3.18c	(2,94) 33.55***	2.15	2.50	2.22
		SD	0.57	0.79	0.98		0.90	0.97	1.02
	FAIM	M	2.60a	2.80a	3.35b	(2,78) 13.98***	2.03	2.09	1.85
		SD	0.96	1.03	0.89		1.03	0.86	0.95

Static	DD	M	2.08a	2.68b	3.30c	(2,70) 37.48***	2.36	2.14	2.11
		SD	0.77	0.94	0.94		1.07	0.95	1.08
	FAIM	M	2.23a	2.81b	3.26c	(2,78) 19.71***	2.57	2.42	2.55
		*SD*	0.82	0.95	0.86		1.01	1.06	1.09

Static = Picture of the apex of the laughter event. Dynamic = Full video animation. DD = Duchenne Laughter. FAIM = Laughs with added frowning as intensity marker. Low = Low Intensity. Medium = Medium Intensity. High = High Intensity. Indexes = a < b < c. ***p < 0.001.

**Table 2** shows the results of the repeated measures ANOVA with the intensity rating as dependent variable and the three intensity stages (low, medium, high) of the two laughter types as repeated measures factor. Also, *post hoc* tests for the intensity ratings within each presentation mode and the two animation types (Duchenne laughs and laughs with eyebrow-lowering furrows) are reported, showing the gradual increase in perceived laughter intensity according to the a priori assigned stimuli intensities.

Convergent with Study 1, **Table 2** shows that the manipulation of the intensity stages in the animations was successful: with increasing laughter intensity, the intensity ratings increased (low < medium < high), *F*(2,300) = 94.46, *p* = 0.001, η_p_^2^ = 0.386. This was true for both presentation modes and animation types. No interaction occurred, *F*(6,300) = 1.51, *p* = 0.174, and the groups did not differ, *F*(3,150) = 2.18, *p* = 0.092; confirming hypothesis H5. In line with the hypothesized knowledge transfer effect, the static presentation did not lead to lower intensity ratings for the high intensity laughs with eyebrow-lowering furrows compared to the Duchenne laugh anymore, once participants had seen the dynamic animations before.

Next, the high intensity laughs (Duchenne and laughs with eyebrow-lowering furrows) were tested for differences in perceived maliciousness in the static and dynamic presentation modes (H6). Hypothesis H6 assumed that the rating bias of eyebrow-lowering frowning as being malicious should disappear in the rating of the presented static stimuli when participants saw the dynamic stimuli first. The means and standard deviations are reported in **Table 2**. An ANOVA with the maliciousness rating as dependent variable and the two animations (Duchenne laughs, laughs with eyebrow-lowering furrows) in two presentation modes (static, dynamic) as grouping factor was computed (see **Table 2**), yielded a significant main effect, *F*(3,158) = 2.77, *p* = 0.044, η_p_^2^ = 0.051. Two planned contrasts (see Winer et al., 1991) disconfirmed H6: the dynamic presentation led to lower maliciousness ratings than the static presentation mode in high intensity laughs (*p* = 0.045 one-tailed) and in laughs with eyebrow-lowering furrows, the static presentation led to higher maliciousness ratings than the dynamic presentation (*p* = 0.005).

### DISCUSSION

Extending the findings of Study 1, Study 2 showed that when participants were confronted with videos and pictures (i.e., received the information from both, the dynamic and the static presentation modes), the perceived intensity did not differ anymore in dependence of the presentation mode: Both, the dynamic and the static presentation led to a linear increase in intensity from the low intensity to the high intensity laugh (in both types of animations), in line with H5. After having seen the videos, participants rated the intensity of the static stimuli congruent to the a priori assigned intensity, independent of whether a frown was present or not. This result might be due to two reasons a) the learning effect of seeing the dynamic stimuli first (transfer of the dynamic knowledge to the static stimuli) and b) the influences of the laughter sound on the perception of intensity (as the laughter sounds were omitted in Study 2 to account for its influences). However, H6 on the perception of maliciousness was not supported: even after having seen the videos (and having processed the full information on the laughter stimuli), participants still rated the maliciousness of statically presented laughs higher (in general, as for laughs with eyebrow-lowering furrows in particular). This is replicating the results of Study 1 and indicates that providing participants with the dynamic laughter information does not diminish the perception of eyebrow-lowering frowning as being malicious in static laughter stimuli.

## GENERAL DISCUSSION

The aim of the present research was to investigate the perception of maliciousness and laughter intensity in intense Duchenne laughter and laughter with added eyebrow-lowering frowning as intensity marker. The findings of two perceptual studies shed light on the emergence of the two competing postulates on the perception of eyebrow-lowering frowning in intense laughter: although eyebrow-lowering frowning is encoded in intense laughs (as Darwin, 1872 and Niewiadomski et al., 2012 suggested), decoding studies utilizing still pictures found eyebrow-lowering frowns to be related to negative emotions and maliciousness, not intensity (see Ruch et al., 2013). The present studies suggest that this is mainly an effect of the presentation mode. Nevertheless, the altering of the perception cannot solely be overcome by presenting participants with both presentation modes in sequence: at least for the rating of maliciousness, the bias toward higher perceived maliciousness of static laughs with a frown persisted, whereas no more differences in the ratings of intensity were found anymore. The preview of the dynamic stimuli might have facilitated the perception of eyebrow-lowering frowning as a feature of laughter intensity, while not influencing the rating of malice as much.

Although the current approach allowed for a fine-grained manipulation of the facial laughter features, one limitation is that the pre-selected and manipulated laughs did not represent the heterogeneity of naturally occurring joyful laughter displays. The laughter of only one subject was utilized, which on one hand decreased the representativeness, but on the other hand kept “personal laughing styles” constant. This control for individual laughter styles (that might go along with specific facial and vocal features) would be problematic, if laughs from different subjects were mixed at different stages of laughter intensity, as then, the qualitative feature changes with increasing intensity would be mixed with the feature changes inherent in the “laughing style” of the subject. For this reason, the selection of laughs was limited to one subject, keeping the “laughter style” constant. Thus, the choice of laughs differed in the perceived laughter intensity (assigned by the raters in the AVLC corpus) and the intensity of facial actions (as coded by FACS experts), which was most important for the investigation of the current research question.

A second limitation targets the influence of the laughter vocalization on the rater’s judgment, which was not explicitly investigated. In Study 1, the presence of the laughter sound in the video animations might have increased the difference between the static and the dynamic stimuli in terms of intensity of the displays (and also the other ratings), as an additional source of information was present in the video animation compared to the pictures. Still, the presence of laughter sound could not have influenced differences between Duchenne laughs and laughs with eyebrow-lowering furrows at converging stages of intensity, as the laughter sounds were the same for the respective laughs, only the facial animations differed (i.e., AU4 added). Although the audio might have an impact on the rating differences between dynamic and static stimuli, its effect on stimuli with modified facial actions (i.e., Duchenne laugh vs. corresponding laugh with eyebrow-lowering furrows) was constant.

A third limitation concerns the adding of the eyebrow-lowering frowning: for the laughs with eyebrow-lowering furrows, the action of the eyebrow-lowering frown was matched to the action of the zygomatic major muscle (AU12) and was therefore present whenever an AU12 was visible. In naturally occurring laughter, the AU4 might only come in at certain stages of intensity of the AU12 (i.e., at marked or extreme intensity; D or E intensity in the FACS) and may not be matched to the intensity of the smiling (AU12). Therefore, future studies should investigate the decoding of stimuli where the eyebrow-lowering frowning (AU4) only occurs at high intensity levels of the AU12, and with both, the eyebrow-lowering frown of a convergent and a discordant intensity to the AU12. Also, natural intense laughs should be encoded for the frequency of eyebrow-lowering frowning (AU4) and possible moderating factors of the occurrence (i.e., personal laughing style) investigated. A pilot analysis of laughs from the AVLC corpus (Urbain et al., 2009) showed that out of 22 randomly sampled intense laughs, nine contained a frown while 13 did not (assessed by the FACS). Unfortunately, the corpus does not provide information on the laughers’ personality.

A forth limitation concerns the investigated modalities. The current study solely focused on facial features of laughter. Vocal and body features were neglected. Still, de Gelder and van den Stock (2011) reported that whole body expressions have an additive value to facial features in the interpretation of emotion expressions. Furthermore, in intense positive and negative emotions (winning or loosing) presented in still pictures, the body has been found to be more important than the face for the correct decoding of the emotion valence (Aviezer et al., 2012). This converges with the conclusions for the presentation modes in the current study: also in the static stimuli utilized by Aviezer et al. (2012), the important dynamic information for the face was missing (as in the static pictures utilized in this approach compared to the dynamic video presentations). Therefore, the decoding of emotion valence was based on incomplete information and might be erroneous, as the results of Aviezer et al. (2012) suggested. In their study, the providing of additional body expressions enhanced the correct decoding through the additional information on the ambiguous facial stimuli (and therefore compensating for the lack of dynamic information). Instead of varying the presentation mode, future studies might also manipulate the in- or exclusion of full body expressions in laughter alongside the facial expressions, to investigate the decoding of intense joyful laughter. A fifth limitation concerns the use of only one (female) avatar, as the facial features of the avatar might interact with the manipulated facial expressions and may impact on the perception (i.e., frowning may look more or less malicious on a particular avatar). Also, the gender of the avatar could possibly influence the ratings. Thus, future studies might utilize avatars of different gender and facial features to increase the ecological validity of the results.

More generally, the current findings also need consideration in the portrayal of laughter by virtual agents. The adequate communication of emotions has been postulated a necessary condition of functioning human-virtual character interactions (see Niewiadomski et al., 2012; Balzarotti et al., 2014). Thus, if frowning alters the perception of a whole laughter expression in certain presentation modes, it can consequently corrupt the interaction with (or evaluation of) a virtual character. For example, confusing an intense joyful laugh with a malicious laugh in a static presentation might lead to the perception of being laughed at or ridiculed by the virtual character. Also, it might lead to a misunderstanding of the further context (i.e., a given task) the laughter is embedded in. Furthermore, as laughter has been postulated to be a marker of several facets of positive emotions (for example Platt et al., 2013 found laughter to occur in *schadenfreude*, amusement, excitement, relief), the present results also suggest implications for research on positive emotions. Thus far, laughter was considered a unitary facial configuration, consisting of the DD plus open mouth (relaxed jaw) and a vocalization, convergent to Duchenne smiling (which has also been postulated as a unitary configuration). The current findings suggest that Darwin’s notion of qualitatively different features of intense laughter should be looked at more closely in encoding studies of natural laughs, as intense laughs might entail facial displays that extend the Duchenne Display definition (but are still indicators of joy). Also in the EMFACS (Ekman et al., 2002), joy is determined solely through the Duchenne markers and any additional AU beyond the Duchenne display leads to an exclusion from the display as being a joy indicator, including intense joyful laughs entailing a frown. Hence, it might be promising for future research on positive emotions to consider laughs that contain eyebrow-lowering frowns in order to gain more understanding of the expression of intense joy through laughter. Recently, Matsumoto and Hwang (2014) found evidence for other facial expressions of emotions beyond the (EM-) FACS prototypes could be accurately judged and thus have emotion signal value (i.e., subtle emotion expressions with low display intensities, display variants including only some of the critical AUs for a display or displays beyond the original definitions by Ekman et al. (2002). It is thus assumed that also for expressions at the extreme end of the intensity spectrum, qualitative feature changes should be more closely examined.

Although research on laughter is increasing, comparatively little is known about the en- and decoding of laughter. There is yet no agreement on a laughter typology (see Ruch et al., 2013) and the present results suggest that even within established laughter types, qualitative feature changes may occur in accordance with different intensity stages and should be more closely investigated and considered in future research.

## Conflict of Interest Statement

The authors declare that the research was conducted in the absence of any commercial or financial relationships that could be construed as a potential conflict of interest.

## Footnotes

1 Other features of laughter such as respiration, head and body movements, circulation, lacrimation, and posture changes have received only little attention (e.g., Hall and Allin, 1897; Wallbott, 1998) and such features have only recently started to find consideration in the literature (e.g., Mancini et al., 2012).

2 Within-subject changes for the ratings of Duchenne laughs and laughs with eyebrow-lowering furrows can be seen in **Figure 3**.
